# Distinct Contribution of the HtrA Protease and PDZ Domains to Its Function in Stress Resilience and Virulence of *Bacillus anthracis*

**DOI:** 10.3389/fmicb.2019.00255

**Published:** 2019-02-18

**Authors:** Ma’ayan Israeli, Uri Elia, Shahar Rotem, Hila Cohen, Avital Tidhar, Adi Bercovich-Kinori, Ofer Cohen, Theodor Chitlaru

**Affiliations:** Department of Biochemistry and Molecular Genetics, Israel Institute for Biological Research, Ness Ziona, Israel

**Keywords:** *Bacillus anthracis*, HtrA protease, proteolytic domain, PDZ domain, auto-processing, virulence, stress sensitivity, anthrax

## Abstract

Anthrax is a lethal disease caused by the Gram-positive spore-producing bacterium *Bacillus anthracis*. We previously demonstrated that disruption of *htrA* gene, encoding the chaperone/protease HtrA_BA_ (High Temperature Requirement A of *B. anthracis*) results in significant virulence attenuation, despite unaffected ability of Δ*htrA* strains (in which the *htrA* gene was deleted) to synthesize the key anthrax virulence factors: the exotoxins and capsule. *B. anthracis* Δ*htrA* strains exhibited increased sensitivity to stress regimens as well as silencing of the secreted starvation-associated Neutral Protease A (NprA) and down-modulation of the bacterial S-layer. The virulence attenuation associated with disruption of the *htrA* gene was suggested to reflect the susceptibility of Δ*htrA* mutated strains to stress insults encountered in the host indicating that HtrA_BA_ represents an important *B. anthracis* pathogenesis determinant. As all HtrA serine proteases, HtrA_BA_ exhibits a protease catalytic domain and a PDZ domain. In the present study we interrogated the relative impact of the proteolytic activity (mediated by the protease domain) and the PDZ domain (presumably necessary for the chaperone activity and/or interaction with substrates) on manifestation of phenotypic characteristics mediated by HtrA_BA_. By inspecting the phenotype exhibited by Δ*htrA* strains *trans*-complemented with either a wild-type, truncated (ΔPDZ), or non-proteolytic form (mutated in the catalytic serine residue) of HtrA_BA_, as well as strains exhibiting modified chromosomal alleles, it is shown that (i) the proteolytic activity of HtrA_BA_ is essential for its N-terminal autolysis and subsequent release into the extracellular *milieu*, while the PDZ domain was dispensable for this process, (ii) the PDZ domain appeared to be dispensable for most of the functions related to stress resilience as well as involvement of HtrA_BA_ in assembly of the bacterial S-layer, (iii) conversely, the proteolytic activity but not the PDZ domain, appeared to be dispensable for the role of HtrA_BA_ in mediating up-regulation of the extracellular protease NprA under starvation stress, and finally (iv) in a murine model of anthrax, the HtrA_BA_ PDZ domain, was dispensable for manifestation of *B. anthracis* virulence. The unexpected dispensability of the PDZ domain may represent a unique characteristic of HtrA_BA_ amongst bacterial serine proteases of the HtrA family.

## Introduction

*Bacillus anthracis* (*B. anthracis*), the etiological cause of the lethal anthrax disease is a spore-forming Gram-positive bacteria. In nature, *B. anthracis* exists as spores which exhibit notorious environmental resilience, and which are the infective form of the bacterium. The animals more frequently affected by the disease are wild or domesticated mammalian herbivores which contract the lethal spores while grazing. Upon infection of a host (cutaneous, gastro-intestinal or respiratory), the metabolically inert spores germinate into fast-dividing toxin-producing bacilli. Human cases of anthrax were frequent in the past due to contact with contaminated animal products or with carcasses of anthrax-succumbed animals. As of today, these cases are extremely rare in the Western world and the interest in the disease stems mainly from the potential intentional malicious use of *B. anthracis* spores as a bio-weapon (for reviews see Koehler, 2009; Chitlaru et al., 2011a).

The lethality of anthrax has been attributed to three main aspects of *B. anthracis* pathogenesis: the activity of the bacterial exotoxins, the anti-phagocytic role of its polyglutamate capsule and the remarkable proliferous nature of the bacteria in the host. This latter aspect of *B. anthracis* pathogenicity suggests that the pathogen excels in exploiting nutritional resources available in the host and is highly adapted to cope with stress constraints encountered in the course of infection. *B. anthracis* secretes two exotoxins, Lethal Toxin (LT) and Edema Toxin (ET) composed of binary combinations of the three proteins: Protective Antigen (PA), the non-harmful subunit of both toxins playing the essential role of binding to a receptor on the surface of host target cells and mediating the intracellular translocation of the lethal subunits of the toxin complex, Lethal Factor (LF), a zinc protease which together with PA forms the exotoxin LT (Klimpel et al., 1994) and Edema Factor (EF) an adenylate cyclase which together with PA constitutes the exotoxin ET, (Leppla, 1982; Lacy and Collier, 2002; Collier, 2009). The three components of the toxin, are encoded by genes located on pXO1, one of the two virulence plasmids naturally harbored by *B. anthracis*. A second well-established virulence factor is represented by a polyglutamate anti-phagocytic capsule synthesized by enzymes encoded by genes located on the second native plasmid pXO2. Anthrax is acknowledged as a toxinogenic disease, owing to the lethality of pure toxin preparations and pivotal role of the toxins in *B. anthracis* virulence, yet, during infection, *B. anthracis* secretes a large number of proteins, many of which bear biological functions indicative of a role in the onset and progression of the disease (Ariel et al., 2002, 2003; Chitlaru et al., 2006, 2007). As of today, a number of proteins, other than the classic toxins, have been suggested to play an essential role during *B. anthracis* infection, based on the attenuated virulence of null mutants entailing targeted disruption of specific genes (Chitlaru et al., 2011a; see Chitlaru et al., 2016 for a list and discussion of reported *B. anthracis* attenuating mutations).

**FIGURE 1:**
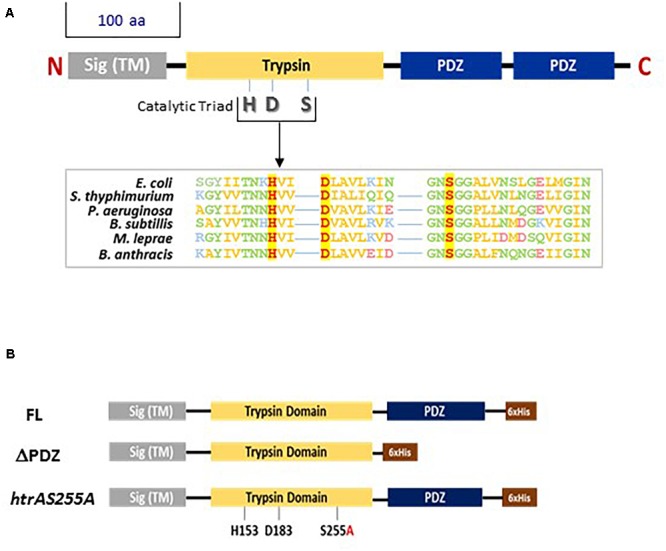
**(A)** Schematic representation of bacterial serine proteases of the HtrA family. The highly conserved domains of the HtrA serine proteases are indicated. Sig (TM)-signal sequence and *trans*-membrane domain; Trypsin-the proteolytic domain. Bacterial HtrA proteases may exhibit one or two PDZ domains. The export signal peptide, trypsin and unique PDZ domain of HtrA_BA_ as well as the H153, D183, and S255 residues forming the catalytic triad of the proteolytic domain were unambiguously identified by alignment with amino-acid sequences of 30 different bacterial serine proteases of the HtrA family using the T-coffee multiple sequence alignment program (Notredame et al., 2000; http://tcoffee.crg.cat). Similar alignments was previously documented (for example, Pallen and Wren, 1997; Kim and Kim, 2005; Singh et al., 2011). Boxed amino-acid sequence alignment: the high sequence conservation around the catalytic H, D, and S residues (shown in red with yellow background) is exemplified for six different bacterial HtrA serine proteases (hydrophobic residues are shown in yellow; polar non-charged residues are shown in green; positively charged residues are shown in blue; negatively charged residues are shown in pink). The respective bacteria are indicated on the left of the alignment. **(B)** Schematic description of the three forms of HtrA_BA_ used in *trans*-complementation experiments for determining the role of the PDZ domain and the proteolytic activity of HtrA_BA_: full-length (FL), truncated (ΔPDZ) or mutated (*htrA*S255A). The *trans*-complementation HtrA_BA_ forms were engineered to exhibit a C terminal stretch of 6 histidine residues (6 × His, brown box) enabling zinc-affinity purification and Western blot visualization using anti-His antibodies.

For all organisms, quality control of protein synthesis is a vital activity. One central player in the context of protein quality control is represented by the HtrA (High Temperature Requirement A) family of serine proteases, which are structurally and functionally conserved across a wide range of evolutionary distinct phylogenetic classes both in prokaryots and eukaryots (reviewed by Clausen et al., 2011; Hansen and Hilgenfeld, 2013; Skórko-Glonek et al., 2013; Backert et al., 2018). HtrA proteins exhibit the dual biological activities of chaperones and proteases (Pallen and Wren, 1997; Spiess et al., 1999; Clausen et al., 2002). HtrA proteins exhibit a characteristic structure (see Figure 1A), composed of an N-terminal serine protease domain and at least one C-terminal PDZ domain that recognizes substrates and in some cases activates the protease function (Wilken et al., 2004; Kim and Kim, 2005). The proteolytic domain entails, among other, the conserved catalytic serine residue whose integrity is essential for proteolytic activity of all serine proteases of the HtrA family. Previous studies evidenced that often the bacterial HtrA characteristic N terminal protease domain (often referred to as a trypsin domain) and the C terminal PDZ domains distinctly impact the proteolytic and chaperone activities of the protein resulting in distinct effects on the phenotypic characteristics of the bacteria (see for example Bæk et al., 2011). While these domains are present in HtrA orthologs of all bacteria (schematically described in Figure 1A), the mutual interactions between the domains affecting their specific activities, in particular the impact of the PDZ domain on the proteolytic activity and substrate specificity, seem to differ among various HtrAs (Kim and Kim, 2005; Clausen et al., 2011; Hansen and Hilgenfeld, 2013; Skórko-Glonek et al., 2013). In addition to the trypsin and the PDZ domains, bacterial HtrAs may exhibit N-terminal canonical Sec export-signal peptides (Tsirigotaki et al., 2017), *trans*-membrane domains (which often enable the membrane localization of the protein) and self-processing domains (which were identified in a number of HtrA and promote self-removal of the N-terminal region of the protein, e.g., Albrecht et al., 2018). In *Escherichia coli* and *B. subtilis*, the HtrA family of proteases are important for the survival of the bacteria under different stress regimens (Pallen and Wren, 1997; Clausen et al., 2002; Antelmann et al., 2003). In addition, in Gram-positive bacteria, the HtrA chaperones/proteases are closely associated with the SecA membrane-translocation machinery suggesting that their targets are constituted by secreted proteins (Tjalsma et al., 2004). In some cases, HtrA was invoked as being directly involved in the proteolytic processing or secretion of specific virulence-associated proteins such as SpeB and hemolysin in *Streptococcus pyogenes* (Lyon and Caparon, 2004; Cole et al., 2007), *Bordetella pertussis* toxin S1 (Vitikainen et al., 2005) and possibly adhesin P1 of *Streptococcus mutans* (Crowley et al., 2008). In other Gram-negative pathogens, such as *Helicobacter pylori*, HtrA was shown to facilitate virulence manifestation by direct proteolysis targeting host proteins such as E-cadherin (Hoy et al., 2010; Tegtmeyer et al., 2016).

High through-put genomic/proteomic/serologic surveys of *B. anthracis* (reviewed in Chitlaru and Shafferman, 2009; Shafferman et al., 2010; Chitlaru et al., 2011a) showed that HtrA belongs to a class of exposed immunogenic putative-vaccine candidates. *B. anthracis* HtrA (HtrA_BA_), which is located both in the membrane as well as in the secretome of the bacteria, is encoded by the unique monocistronic *htrA* gene (NCBI locus tag BA6330 in the *B. anthracis* Ames-ancestor reference strain). HtrA_BA_ was shown to be present in the circulation of infected animals and consequently could serve as a potential anthrax early disease-biomarker (Sela-Abramovich et al., 2009). Disruption of the *htrA* gene in toxinogenic *B. anthracis* Vollum (pXO1^+^; pXO2^+^) or Sterne (pXO1^+^; pXO2^-^) strains resulted in a dramatic attenuation in the guinea pig, murine and rabbit models of anthrax (Chitlaru et al., 2011b, 2016). Previous studies of these *htrA*-disrupted *B. anthracis* strains (to be referred from hereon as Δ*htrA*, Chitlaru et al., 2010, 2011b, 2016, 2017) have demonstrated that while abrogation of HtrA_BA_ synthesis does not affect the *in vitro* growth of the bacteria in culture, it results in a complex phenotype exhibiting the following characteristics: (i) *in vitro* increased sensitivity to stress (heat, oxidative, salt, and ethanol), (ii) failure to up-regulate the NprA secreted protease under nutritional deprivation, (iii) inability to assemble the typical *B. anthracis* S-layer (in strains cured of the pXO1 and pXO2 native plasmids), (iv) propagation delay in a macrophage-infection assay (suggested to reflect the increased sensitivity to oxidative stress). The significant attenuation of virulence of *B. anthracis* Δ*htrA* strains suggested that HtrA_BA_ is essential for manifestation of *B. anthracis* pathogenicity and accordingly, represents a novel *B. anthracis* virulence determinant. Consequently, *htrA*-gene disruption in the non-capsular Sterne-strain served for the development of an efficacious and safe next-generation live attenuated anthrax spore-vaccine (Chitlaru et al., 2016, 2017). The role of HtrA_BA_ in promoting tolerance to various stress stimuli, similar to that observed in a variety of Gram-positive and negative bacteria (Skórko-Glonek et al., 2013; Backert et al., 2018) may represent the basis for the dramatic virulence-attenuation of the *htrA* disrupted bacteria, reflecting the inability of the mutated cells to cope with stress conditions encountered in the host. Of note, resilience to oxidative stress in the course of infection is considered to represent an important feature of pathogenic bacteria in general, and *B. anthracis* in particular (Welkos et al., 2011).

In the current report we inspect the contribution of the protease and PDZ-domains of *B. anthracis* HtrA to its function (as evidenced by the phenotype associated with its disruption). Accordingly, muteins exhibiting either deletion of the PDZ domain or mutational abrogation of the proteolytic activity were expressed in the Δ*htrA* (*htrA*-disrupted) strains enabling assessment of their ability to *trans*-complement their defective phenotype. Additionally, *B. anthracis* mutated strains exhibiting chromosomal allele modifications altering either the proteolytic domain or deletion of the PDZ domain were generated. The data suggest that the proteolytic activity of HtrA is essential for its self-processing and subsequent export of HtrA_BA_ in to the extracellular *milieu*. The PDZ domain appears to be dispensable for this autolytic process. Furthermore, the PDZ domain was dispensable for most of the inspected functions related to stress resilience as well as for the involvement of HtrA_BA_ in the assembly of the bacterial S-layer. On the other hand, PDZ was essential for the HtrA_BA_ role in mediating up-regulation of the extracellular protease NprA under starvation stress. In a murine model of anthrax, it is shown that the HtrA_BA_ PDZ domain, is not essential, while the proteolytic activity is necessary for manifestation of *B. anthracis* virulence.

## Materials and Methods

### Bacterial Strains, Media and Growth Conditions

*Bacillus anthracis* strains used in this study are listed in Table 1. *E. coli* strains, are detailed in Supplementary Table S1. *B. anthracis* were cultivated at 37°C, in FAG broth (3.3% tryptone, 2% yeast extract, 0.74% NaCl, 0.4% KH_2_PO_4_, 0.8% Na_2_HPO_4_, 2% glycerol, pH 8), in Brain-Heart Infusion (BHI, DIFCO/Becton Dickinson) or in NBY low-nutrient content medium [0.8% (w/vol) Nutrient broth (Difco), 0.3% Yeast extract (Difco) and 0.5% Glucose]. *E. coli* were cultivated at 37°C, in Luria-Bertani (LB, Difco). LB-agar was used as solid broth. *E. coli* strains were used for plasmid construction. Antibiotic concentrations used for the selection in LB agar/broth were: for *E. coli* strains, ampicillin (Amp, 100 μg ml^-1^); for *B. anthracis* strains, Chloramphenicol (Cm, 5 μg ml^-1^), kanamycin (10 μg ml^-1^), and erythromycin (5 μg ml^-1^).

**Table 1:** *Bacillus anthracis* strains used in the present study.

	*B. anthracis* Strain	Nomenclature throughout the article	Significance; Reference
(1)	Sterne	Sterne	Toxinogenic Non-capsular (pXO1^+^, pXO2^-^); (Turnbull, 1991)
(2)	Sterne Δ*htrA*	SΔ*htrA*	Sterne strain with a deleted *htrA* gene; (Chitlaru et al., 2016)
(3)	ΔVollum	ΔV	Non-toxinogenic and non-capsular plasmid-cured (pXO1^-^, pXO2^-^) derived from the ATCC14578 Vollum virulent strain; (Chitlaru et al., 2010, 2011b)
(4)	ΔVollumΔ*htrA*	ΔVΔ*htrA*	ΔV strain with a deleted *htrA* gene; (Chitlaru et al., 2010, 2011b)
(5)	ΔVollum*htrA_D_*_PDZ_	ΔV*htrA*_DPDZ_	ΔV strain containing an *htrA* allele with a deleted PDZ domain
(6)	ΔVollum*htrA*_S255A_	ΔV*htrA*_S255A_	ΔV strain containing an *htrA* allele with a S255A point mutation
(7)	Sterne Δ*htrA/HtrA*	SΔ*htrA/HtrA*	Sterne Δ*htrA trans*-complemented with a full-length *htrA* gene
(8)	Sterne Δ*htrA/HtrA*ΔPDZ	SΔ*htrA/HtrA*ΔPDZ	Sterne Δ*htrA trans*-complemented with an *htrA* gene lacking the PDZ domain
(9)	Sterne Δ*htrA/HtrA*S255A	SΔ*htrA/HtrA*S255A	Sterne Δ*htrA trans*-complemented with an *htrA* S255A gene
(10)	ΔVollumΔ*htrA/HtrA*	ΔVΔ*htrA/HtrA*	ΔVΔ*htrA trans*-complemented with a full-length *htrA* gene
(11)	ΔVollumΔ*htrA/HtrA*ΔPDZ	ΔVΔ*htrA/HtrA*ΔPDZ	ΔVΔ*htrA trans*-complemented with an *htrA* gene lacking the PDZ domain
(12)	ΔVollumΔ*htrA/HtrA*S255A	ΔVΔ*htrA/HtrA*S255A	ΔVΔ*htrA trans*-complemented with an *htrA* S255A gene

*Bacillus anthracis Sterne (strain 1) is a non-capsular vaccine strain from the IIBR collection. Strains 5–12 were generated in the current study. Strains 7–12 are trans-complemented strains in the back-ground of Sterne ΔhtrA (strains 7–9) or ΔVollumΔhtrA (strains 10–12). See also Figure 1B for schematic description of the experimental approach using the strains listed in the table.*

### Plasmid and Strain Construction

Plasmids and oligonucleotide primers used in this study are summarized in Supplementary Table S1. The oligonucleotide primers were designed according to the genomic sequence of *B. anthracis* Sterne strain. Point mutations were introduced using QuikChange site-directed mutagenesis kit (Statagene/Agilent). Prior to transformation into *B. anthracis* all plasmids were propagated in the methylation deficient *E. coli* strain *dam^-^dcm^-^*. *B. anthracis* cells were electrotransformed as described (Cohen et al., 2000). To express HtrA_BA_ as C-terminal His tag protein under *amy* promoter, the *htr*A complete gene was cloned as a *Sna*BI/*Bam*HI-digest of PCR product (BA531 and BA819C primers), replacing the *pag*A gene in the previously described vector pASC-α (Cohen et al., 2000). Similary, a 900 bp segment encoding the N terminal 300 amino acids of HtrA_BA_ was cloned into pASC-α (BA531 and BA820C primers) to generate the ΔPDZ version of HtrA_BA_. To generate a catalytic mutant of HtrA_BA_, the conserved Ser255 (on the basis of alignment with the amino-acid sequences of a variety of HtrA proteases) was mutated to an alanine residue. Oligonucleotide-directed mutagenesis was performed using primers BA823 and BA824C on plasmid pASC-α/HtrA, to generate pASC-α/HtrA_S255A_. The vectors used for disruption of *htrA* gene by allelic replacement were previously described (Chitlaru et al., 2017). pEGS-N’HtrA was used to disrupt the C’ terminus of *htrA* gene. The plasmid was constructed in four steps: (i) The vector pEGS-cya (Supplementary Table S1, Levy et al., 2012) was digested with *Spe*I and *Not*I; (ii) a *Spe*I –*Not*I restriction fragment of the *htrA* N’terminus segment (900 bp) derived by PCR using BA827 and BA828C was cloned into the vector from step (i); (iii) the vector generated in step (ii) was digested with *Asc*I and *Spe*I; (iv) a *Asc*I- *Spe*I restriction fragment of the *htrA* 5′UTR segment (900 bp) derived by PCR using BA829 and BA830C was cloned into the vector from step iii. pEGS-HtrA_S255A_ was used to disrupt the conserved Ser255 of *htrA* gene. The plasmid was constructed in two steps: (i) The vector pEGS-*cya* was digested with *Asc*I and *Not*I; (ii) a *Asc*I –*Not*I restriction fragment of the *htrA*_S255A_ derived by PCR using BA827 and BA831C and pASC-α/HtrA_S255A_ as template was cloned into the vector from step (i).

### Stress Sensitivity Agar Dilution Drop Assay

WT parental ΔV, ΔVΔ*htrA* and *trans*-complemented strains ΔVΔ*htrA/HtrA*, ΔVΔ*htrA/HtrA*ΔPΔZ and ΔVΔ*htrA/HtrA*S255A (see Table 1 for nomenclature of strains used in this study and Supplementary Table S1 for the plasmids used to generate the *trans*-complemented strains), were grown in BHI to mid-log phase, brought to an OD of 1 OD unit and decimally diluted. All OD measurements were performed at a wavelength of 600 nm. Ten ml of each serial dilution was dropped on LB-agar plates containing various concentrations of freshly prepared H_2_O_2_ (final concentration 0.75 or 1.5 mM) or 3% NaCl. Plates were incubated at 37°C or at the indicated temperatures over-night.

### DNA Preparation and Polymerase Chain Reaction (PCR)

Restriction enzymes (Fermentas) and T4 DNA ligase (Promega) were used as recommended by the supplier. Plasmid DNA were extracted from *E. coli* using Wizard Plus SV mini preps (Promega). PCR amplifications were performed using the MyTaq (Bioline) or Expand High Fidelity (Roche) systems. For fast colony screening, each colony was re-suspended in 25 μl PCR mix. PCR products were separated on 1% agarose gel using 1X TAE as running buffer and purified using QIAquick PCR purification kit (Qiagen). DNA sequences were determined with the ABI310 rhodamine termination reaction kit (ABI310 Genetic Analyzer, Applied Biosystems).

### SDS-PAGE and Western Blot Analysis of *B. anthracis* Cultures

Bacterial pellets or bacterial secreted proteins (supernatants) collected from *B. anthracis* cultures [20 h post-inoculation with an over-night starter, at an initial optical density of 0.05 OD (optical density units), were analyzed by SDS-PAGE and Western blotting]. The final concentration of the culture was typically 10 OD (approx. 10^9^CFU/ml) in Fag medium and 3 OD in NBY medium. Typically, the equivalent of 2 × 10^8^ CFU (for analysis of the cell-associated fraction) or proteins secreted by 2 × 10^8^ CFU (for analysis of the secreted material) was loaded per gel lane after 10 min boiling in SDS-load buffer. SDS-PAGE was carried out on 4–12% NuPage Bis-Tris gels (Invitrogen) using Precision Plus Molecular weight markers (Bio-Rad). Western blots were generated using the Nitrocellulose Western iBlot Gel transfer Semi-dry system (Invitrogen). The nitrocellulose membranes were blocked in LiCor blocking buffer for 1 h. at room temperature, and probed with primary antibody overnight at 4°C. The membranes were washed three times for 10 min in PBST (PBS containing 0.05% Tween), probed with secondary antibody for 1 h at room temperature and washed twice. The blots were scanned using the LiCor laser-based image detection method. The following antibodies were used in this study: mouse anti-HtrA (Chitlaru et al., 2011b), mouse anti-His (iii) rabbit anti-S layer (iv) mouse anti-NprA (v) IRDye^®^800CW conjugated goat anti-mouse (vi) IRDye^®^800CW conjugated goat anti-rabbit. Primary and secondary antibodies were used at 1:500 and 1:20,000 dilutions, respectively.

### Expression in *E. coli* and IMAC (Immobilized Metal Affinity Chromatography) Purification of HtrA

To induce expression of HtrA_BA_, an overnight culture in LB with 100 μg/ml ampicillin was diluted 1/100 in 20 ml fresh medium and grown to an optical density (600 nm) of 0.6; 1 mM IPTG (isopropyl-β-D-thiogalactopy-ranoside) was added and the culture continued for 4 h. Cells were harvested by centrifugation (10 min, 10,000 rpm, 4°C), washed once with PBS and pellet was frozen at -70°C until lysis. Cells were lysed on ice in lysis buffer (50 mM NaH_2_PO_4_, 300 mM NaCl, 10 mM imidazole, 0.05% tween20, pH 8.0) containing 1 mg/ml lysozyme, followed by sonication; lysates were cleared by centrifugation (30 min, 10,000g, 4°C). His-tagged HtrA proteins were purified by Ni-NTA magnetic agarose beads (Qiagene, 36111). Non-specifically bound proteins were removed with wash buffer (50 mM NaH_2_PO_4_, 300 mM NaCl, 20 mM imidazole, 0.05% tween20, pH 8.0), and HtrA was eluted with elution buffer (50 mM NaH_2_PO_4_, 300 mM NaCl, 10 mM imidazole, 0.05% tween20, pH 8.0). Eluted proteins were dialyzed against PBS (Slide-A-lyzer 10K dialysis cassettes G2, #807729, Thermo Scientific).

### Proteolytic Activity of HtrA_BA_

Proteolytic activity of HtrA_BA_, HtrA_BA_ΔPDZ or HtrA_S255A_ was determined using casein as substrate as described (Bæk et al., 2011). In brief, reaction mixtures containing 50 mM Tris-HCl (pH 8.0), 1 mg β-casein (Sigma C6905) and 0.4 μM HtrA_BA_ or HtrA_BA_ΔPDZ or HtrA_S255A_ were incubated at 37°C for 18 h and terminated by the addition of 10 μl 4XSDS sample buffer and heating to 100°C for 5 min. samples were separated by SDS-PAGE and stained with GelCode blue stain reagent (Thermo scientific #24590).

### Chaperone Activity of HtrA

Chaperone activity of HtrA_BA_, HtrA_BA_ΔPDZ or HtrA_S255A_ was performed under aggregation-prone conditions as described (Skórko-Glonek et al., 2003; Entzminger et al., 2012). In brief, egg-white lysozyme (Sigma L3790) was adjusted to 100 μM in denaturation buffer (HEPES-buffered saline, HBS [10 mM HEPES, 150 mM NaCl, pH 7.4] with 8 M urea and 50 mM DTT at pH 7.4) and allowed to equilibrate for 6 h. To monitor aggregation, unfolded and reduced lysozyme was rapidly diluted 100-fold into HBS alone or HBS containing HtrA_BA_ at concentration of 1 μM in 96-well plate. Protein aggregation was monitored by absorbance at 360 nm for up to 3 h at room temperature with a SpectroMax spectrophotometer.

### Liquid Chromatography Mass Spectrometry (LC-MS) Mapping of HtrA

Liquid chromatography mass spectrometry of the heavy, light and truncated forms of HtrA_BA_ was performed at the De Botton Protein Profiling Institute of the Nancy and Stephen Grand Israel National Center for Personalized Medicine, Weizmann Institute of Science, Rehovot. In brief, gel bands containing the different forms of HtrA_BA_ were subjected to in-gel pepsin digestion. The resulting peptides were analyzed by nanoflow liquid chromatography coupled to high resolution, high mass accuracy mass spectrometry. The data was processed using Proteome Discoverer version 2.2.0.388, searched against the Uniprot *Bacillus anthracis* Ames ancestor reference strain protein database using both the SequestHT and Mascot search algorithms.

### Infection of Experimental Animals

Outbred ICR mice (20–25 g, Harlan) were infected with vegetative cells of various *B. anthracis* Sterne-derived strains. All Sterne derived strains used in the virulence study, exhibited similar levels of LF, EF, and PA activity, determined by functional assays, as previously described (Israeli et al., 2016; Chitlaru et al., 2017). For infection, bacterial cultures were set in BHI medium by inoculation with an over-night starter at an initial density of 0.05 × OD units. Bacteria were grown for 3–4 h at 37°C, to mid-logarithmic phase, (approximately 2 × ODunits). Bacteria were inspected by microscope for similar chain length of vegetative cells, then centrifuged and re-suspended in PBS at the desired concentration (1 × OD unit = 10^8^ CFU) such that mice were infected SC with 0.1 ml bacterial suspension and serial 10-fold dilutions. A total of 4 mice per dose per strain were used. The remaining bacterial dose suspensions were plated for total viable counts (CFU ml^-1^) to confirm the dose administered to the animals. For the virulence determination, the animals were observed daily for 21 days. The lethal dose required to kill 50% (LD_50_) of the animals was calculated by non-linear fit regression using the GraphPad Prism (version 5.0) statistical analysis software (San Diego, CA, United States). Animal experiments were approved by the IIBR committee for animal research. The IIBR animal-experiment protocol number was M-07-18. The experimental animals were handled according to the National Research Council 1996 Guide for the Care and Use of Laboratory Animals and regulations of the IIBR Animal Use Committee.

## Results

### General Design of the Study

To inspect the contribution of the protease and PDZ-domains of *B. anthracis* HtrA to its function, we generated *trans*-complementation vectors (Figure 1B) expressing three distinct recombinant forms of HtrA_BA_, representing (i) the intact full-length of the protein (FL), (ii) a truncated form in which the PDZ domain is deleted (ΔPDZ), and (iii) an HtrA_BA_ form in which the catalytic serine necessary for proteolytic activity was point-mutated (S255A). The *trans*-complementation versions of HtrA_BA_, tailored to express a C-terminal *6-his-tag* were first expressed in *E. coli*, purified by immobilized metal affinity chromatography (IMAC) and used in assays which probed the proteolytic and chaperone activities, enabling confirmation that these activities are, as expected, differentially affected by the S255A mutation and by the PDZ deletion, respectively (Supplementary Figure S1).

The three forms of the proteins were then expressed in *B. anthracis* Δ*htrA*, to interrogate the contribution of the protease and chaperone activities of HtrA_BA_ in determining the manifestation of major phenotypic features displayed by the Δ*htrA* strains. In addition to this approach of *trans*-complementation of the Δ*htrA* phenotype, an alternative, approach, consisting of direct modification of the chromosomal *htrA* allels, was carried out (Supplementary Figure S2). Accordingly, targeted gene-replacement modifications of the gene resulted in the generation of *B. anthracis* strains entailing total deletion of the *htrA* gene (Δ*htrA*), partial deletion encompassing the PDZ domain (*htrA*_DPDZ_) or point replacement mutation of the catalytic serine residue (*htrA*_S255A_). The various *B. anthracis* strains used in this study, exhibiting either allelic modified versions of the *htrA*_BA_ gene or expressing various *trans*-complementing forms of HtrA_BA_ in the background of the Δ*htrA* strain are detailed in Table 1.

### The Proteolytic Activity but Not the PDZ Domain Is Essential for Self-Processing and Consequent Secretion of HtrA_BA_

**FIGURE 2:**
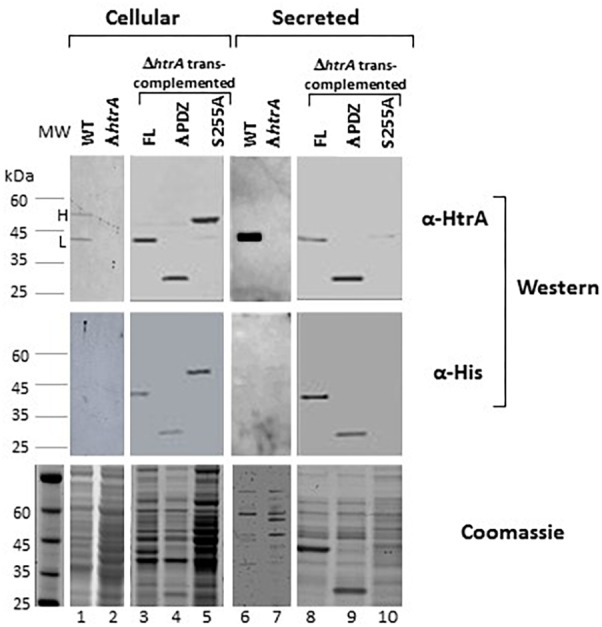
The S255A mutation abrogating the proteolytic activity prevents the N terminus auto-processing and secretion of HtrA_BA_. Western-blot analysis of cellular (lanes 1–5) and secreted (lanes 6–10) proteins of the WT parental ΔVstrain (lanes 1 and 6), ΔVΔ*htrA* (lanes 2 and 7) and the *trans*-complemented strains ΔVΔ*htrA/HtrA* (lanes 3 and 8), ΔVΔ*htrA/HtrA*ΔPDZ (lanes 4 and 9) and ΔVΔ*htrA/HtrA*S255A (lanes 5 and 10). Western blots were probed with anti-HtrA and anti-His antibodies, as indicated. The two forms of HtrA_BA_ are indicated as H (heavy, unprocessed) and light (auto-processed).

Extrachromosomal expression of the full length (FL), ΔPDZ and S255A versions of HtrA_BA_ in *B. anthracis* Δ*htrA* bacteria resulted in biosynthesis of the respective proteins which could be detected by Western-blot analysis in the cellular as well as secreted fractions, as depicted in Figure 2. Typically, the cellular fraction of *B. anthracis* WT cells contains 2 distinctly electrophoretic migrating HtrA bands corresponding to the FL and N-terminal processed protein (Figure 2), while the secreted fraction mainly contains the short processed form (Figure 2, see also Chitlaru et al., 2011b). The 2 forms will be referred hereon as H (heavy) and L (light). The detection of these two forms did not appear to reflect the forms of HtrA before and after the removal of the N-terminal signal peptide in the course of protein export (Tsirigotaki et al., 2017; Freudl, 2018), since the apparent electrophoretic migration-difference between the H and L forms implied the removal of an N-terminal fragment significantly longer than the predicted signal peptide (calculated to span the first 47 amino-acids of the protein, equivalent only to approximately 5 kDa) suggesting that the processing occurs at a location down-stream of the predicted signal-peptide cleavage site. Interestingly, the S255A mutated HtrA_BA_ migrated on SDS-gel as the H form (unprocessed) only. Furthermore, the mutated S255A version was much more abundant in the cellular fraction and almost undetected in its secreted form while the FL and ΔPDZ forms were abundantly present in the secretome (as well as in the cellular fraction). The fact that both anti-HtrA and anti-His antibodies generated similar Western-blot patterns (Figure 2) confirmed that the observed electrophoretic-migration differences stemmed from the processing of the N terminus (note that the His tag is located in the C terminus of the *trans*-complementation HtrA forms, see Figure 1B). These results establish that the proteolytic inactive HtrAS255A protein is retained in the bacteria and fails to be secreted probably due to its inability to process its N-terminus.

**FIGURE 3:**
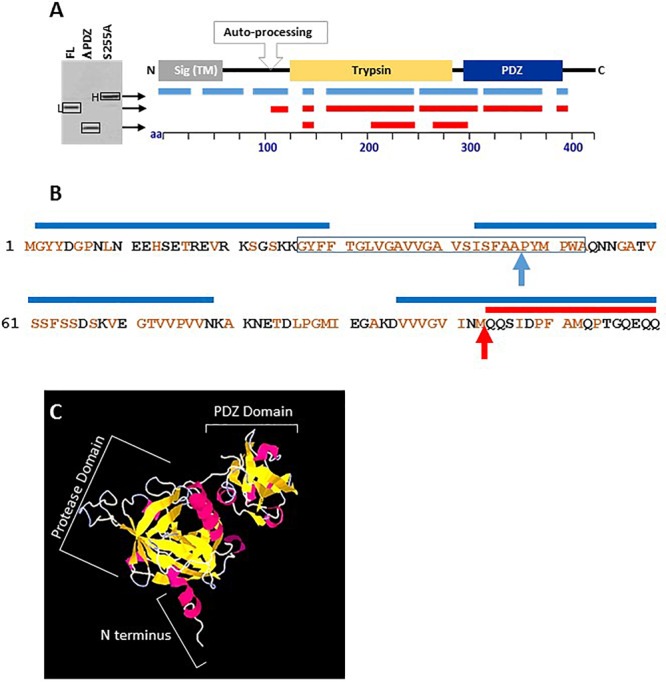
Liquid chromatography mass spectrometry (LC-MS) mapping of the heavy S255A (unprocessed), the light FL (auto-processed) and the truncated ΔPDZ forms of HtrA_BA_. **(A)** The lines under the schematic linear depiction of HtrA_BA_ describe the position of the peptides detected by LC-MS in the respective forms of HtrA_BA_ collected from the SDS-gel as indicated by arrows. Positions of peptides obtained from digestion of the H form are indicated in blue while those from the L form or the ΔPDZ form are in red. **(B)** The amino-acid sequence of the first 120 residues of HtrA_BA_. Brown residues are hydrophobic. The blue lines above the sequence indicate peptides identified by LC-MS in the heavy (H) form of HtrA_BA_S255A. The red line above the sequence indicates the first peptide identified by LC-MS in the light (L) form of HtrA_BA_. The highly hydrophobic *trans*-membrane domain is framed. The blue arrow indicates the predicted site of signal peptide cleavage. The red arrow indicates the predicted site of proteolytic auto-processing. **(C)** High confidence 3D structural modeling of HtrA_BA_ (created by using PHYRE Protein Fold Recognition modeling, Kelley et al., 2015) showing the clear spatial separation of the indicated protease and PDZ domains of the protein and that its first 100 amino acids constitute an exposed protruding domain (marked as N terminus), highly accessible to proteolysis without affecting the general architecture of the protein.

To further confirm that the auto-processing involves removal of the N-terminus and to map the site of the proteolysis, the electrophoretic distinct H and L forms of HtrA_BA_, as well as the ΔPDZ version were inspected by liquid chromatography mass spectrometry (LC-MS) of in-gel pepsin digestion fragments (see section “Materials and Methods”). Digestion of the H and L forms established that peptides originating from the N-terminus of the protein could be detected only in the H form (representing the HtrAS255A mutant) while they were absent from the L form of HtrA_BA_ or the HtrA_BA_ΔPDZ version (Figure 3A). With the exception of the N-terminal region, digestion generated peptides covering most of the proteins. Comparison of the sequences of the N-terminal fragments detected in the H and L forms suggested that the cleavage of the N terminus occurred at position 103 of the protein (based on the detection of a pepsin fragment spanning amino acids 96–129 in the H form while the most N-terminal fragment detected in the L form spans amino acids 104–129). Cleavage at this site is in line with the difference (of about 10 kDa) observed between the electrophoretic migration of the two HtrA_BA_ forms (see detailed N-terminus sequence in Figure 3B). While we cannot accurately establish the exact site of the N-terminal self-proteolysis, it is conceivable that cleavage at this site results in deletion of a highly hydrophobic *trans*-membrane fragment whose removal is necessary for the release of the protein into the medium (squared fragment in Figure 3B). Of note, 3D structural modeling of HtrA_BA_ (using the PHYRE Protein Fold Recognition server, Kelley et al., 2015) established with high confidence that the first 100 amino acids of the protein constitute a highly exposed protruding domain (preceding the protease domain) which appears to be highly accessible to proteolysis without affecting the general architecture of the protein (Figure 3C). This observation supports the notion that the auto-processing occurs around position 100 of the protein.

Taken together, these results provide support for the conclusion that (i) HtrA_BA_ is auto-processing its N-terminus as documented for a variety of bacterial HtrA paralogs (Skórko-Glonek et al., 1995, 2003; MohamedMohaideen et al., 2008; Hershey et al., 2016; Zhu et al., 2017; Albrecht et al., 2018, see section “Discussion”), (ii) that the proteolytic activity (abrogated by the S255A mutation) is essential for this process, (iii) that the N-terminus processing is necessary for efficient secretion of HtrA_BA_, and (iv) finally and most intriguing, the PDZ domain is dispensable for the auto-proteolytic process.

### The Proteolytic Activity but Not the PDZ Domain Is Essential for the Role of HtrA_BA_ in Growth of *B. anthracis* Under Temperature, Oxidative and Salt Stress

**FIGURE 4:**
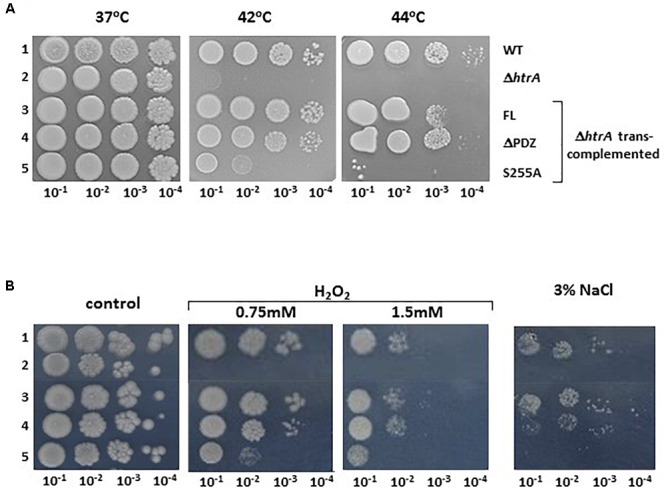
Dilution drop agar-assay of the WT parental ΔVstrain (1), ΔVΔ*htrA* (2), and the *trans*-complemented strains ΔVΔ*htrA/HtrA* (3), ΔVΔ*htrA/HtrA*ΔPDZ (4) and ΔVΔ*htrA/HtrA*S255A (5) under various stress conditions. **(A)** Alleviation of the heat sensitivity phenotype of the Δ*htrA* strain by *trans*-complementation with the FL and ΔPDZ forms of HtrA_BA_. Plates were incubated at the indicated temperatures. **(B)** Alleviation of the H_2_O_2_ and NaCl sensitivity phenotype of the Δ*htrA* strain by *trans*-complementation with the FL and ΔPDZ forms of HtrA_BA_. Control LB-agar plates or LB plates containing the indicated concentrations of H_2_O_2_ or NaCl were incubated at 37°C.

Involvement of HtrA_BA_ in the resilience of the bacteria to stress was demonstrated previously by determining the growth of Δ*htrA* strains under various stress conditions and is considered to represent the main reason for the virulence attenuation associated with *htrA* gene disruption (Chitlaru et al., 2011b, 2016). To determine the relative contribution of the proteolytic or the PDZ domains to the role of HtrA_BA_ in the resilience of the bacteria to stress, Δ*HtrA trans*-complemented with the FL, ΔPDZ or S255A forms of HtrA_BA_ were subjected to a growth dilution drop-assay addressing sensitivity to high temperature, oxidative stress and salt, as depicted in Figure 4 (see also Supplementary Figure S3 for temperature sensitivity complementation using liquid cultures). The results establish that sensitivity to high temperature, hydroxide peroxide and salt exhibited by the *htrA* disrupted strain can be fully alleviated by *trans*-complementation with the FL or ΔPDZ forms but not with the S255A mutated form. The data support the conclusion that the proteolytic activity (abrogated by the S255A mutation) is essential, while the PDZ domain is dispensable for the role of HtrA in stress resilience.

### The Proteolytic Activity but Not the PDZ Domain Is Essential for the Role of HtrA_BA_ in the Biosynthesis of *B. anthracis* S-Layer

**FIGURE 5:**
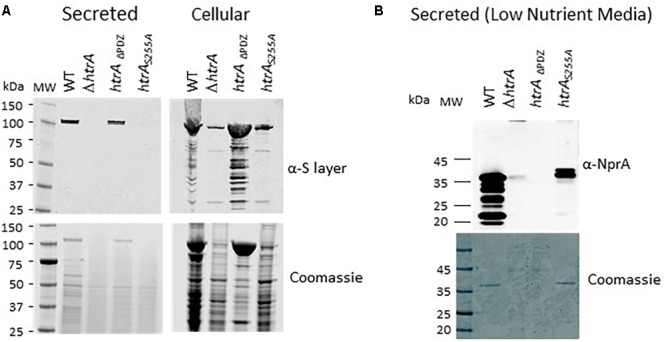
Expression of S-layer proteins and of the NprA secreted protease by the *B. anthracis* parental WT ΔV, the ΔVΔ*htrA* and the ΔV strains exhibiting modified *htrA* alleles ΔV*htrA*_DPDZ_ and ΔV*htrA*_S255A_. **(A)** Western blot analysis of the secreted and cell associated protein fractions collected from BHI cultures of the strains using anti-S layer antibodies. **(B)** Western-blot analysis of the secreted protein fraction collected from low-nutrient NBY cultures of the strains using anti-NprA antibodies.

We have documented in the past that in *B. anthracis* strains cured of the pXO1 and pXO2 native virulence-plasmids (such as the ΔVollum strain), disruption of the *htrA* gene results in the down-modulation of the *B. anthracis* S layer (Chitlaru et al., 2010, 2011b). This phenomenon, occurred post-transcriptionally and affected both the Sap and EA1 proteins which form the bacterial S-layer (Fouet, 2009) both in their cell-associated and secreted forms. Interestingly, this phenomenon was not observed in virulent *B. anthracis* strains (exhibiting the pXO1 virulence plasmid), suggesting that post-translational biosynthesis of the S-layer involves at least two alternative compensatory pathways, one governed by functions encoded in the pXO1 plasmid and another, HtrA-dependent mechanism which consists of chromosomally encoded functions (see Chitlaru et al., 2011b for an extensive discussion of this phenomenon). In the current study, we addressed the question whether the S-layer biosynthesis may be mediated by the mutated ΔPDZ or the S255A forms of HtrA. Accordingly, the level of S-layer proteins in the cell associated and secreted fractions of the wild-type parental (ΔVollum) as well as in the strains exhibiting chromosomally modified alleles ΔVΔ*htrA*, ΔV*htrA*_DPDZ_ and ΔV*htrA*_S255A_ strains was evaluated by Western blot analysis using antibodies recognizing both the Sap and EA1 S-layer proteins. The data depicted in Figure 5A provide evidence that the ΔPDZ form of HtrA is sufficient to mediate the observed effect on S-layer modulation, similar to the effect observed in the parental strain, while the S255A non-proteolytic form of HtrA failed to assist the modulation of the S-layer bio-synthesis. This modulation of S-layer level by the FL or the ΔPDZ forms of HtrA (but not by the S255A mutant) observed in the chromosomally modified strains, was confirmed by the alternative *trans*-complementation approach of the Δ*htrA* strain (Supplementary Figure S4). Based on the data obtained by both approaches it may be concluded that the proteolytic activity of HtrA_BA_ is essential for the S-layer biosynthesis modulation in the *B. anthracis* ΔVollum bacteria while the PDZ is dispensable for this role.

### The PDZ Domain but Not the Proteolytic Activity Is Essential for the Role of HtrA_BA_ in Up-Regulation of the NprA Secreted Protease Under Low-Nutrient Conditions

In low-nutrient media, a prevalent part of the *B. anthracis* secretome is constituted by the secreted protease NprA, which is not expressed in high-nutrient culture and is down-regulated in CO_2_-enriched media (Chitlaru et al., 2006, 2010). The dramatic up-regulation of NprA has been suggested to reflect a response to the depletion of nutrients and consequent requirement for proteolytic activities enabling compensatory exploitation of proteins for survival. We have documented that Δ*htrA* strains do not exhibit up-regulation of the NprA protease in low-nutrient media or following extended (>30 h) culture in rich media (Chitlaru et al., 2010, 2011b). This characteristic of the Δ*htrA* strains was attributed to the role of HtrA in the ability of *B. anthracis* to cope with various stress regimens. The involvement of HtrA in the up-regulation of NprA involved induction of the *npr*A gene transcription, as opposed to the effect of HtrA on S-layer modulation, which occurs post-transcriptionally (see above). In the current study, we addressed the question whether the induction of NprA may be mediated by the mutated ΔPDZ or the S255A forms of HtrA_BA_. Accordingly, the level of NprA in the low-nutrient cultures of the wild-type parental (ΔVollum), as well as the chromosomally modified ΔV Δ*htrA*, ΔV*htrA_Δ_*_PDZ_ and ΔV*htrA*_S255A_ strains was evaluated by Western blot analysis using anti NprA antibodies. The data depicted in Figure 5B provide evidence that the S255A non-proteolytic form of HtrA is sufficient to mediate the observed induction of NprA, while the bacteria expressing the ΔPDZ form of HtrA_BA_ did not exhibit induction of NprA. Thus, it may be concluded that the PDZ domain is essential for the induction of NprA in *B. anthracis* while the proteolytic activity of HtrA is dispensable for this role.

### The Proteolytic Activity but Not the PDZ Domain Is Essential for the Role of HtrA_BA_ in Manifestation of *B. anthracis* Virulence

**FIGURE 6:**
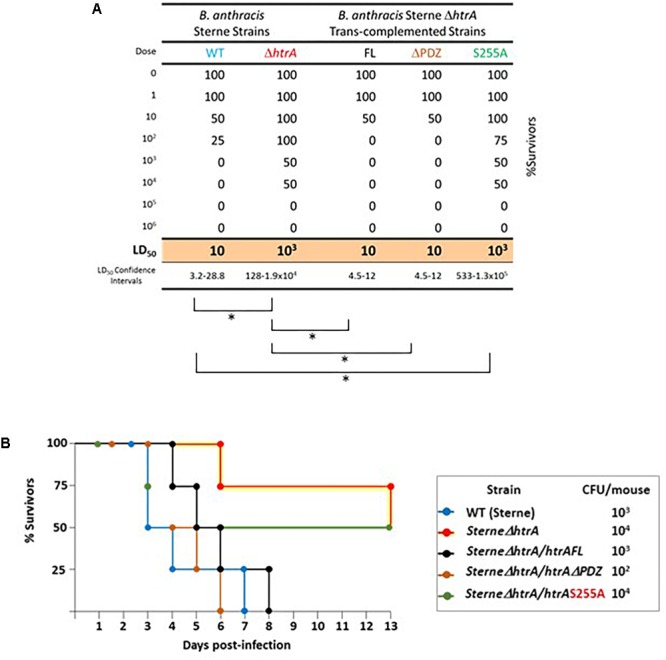
Virulence of *B. anthracis* Sterne, SΔ*htrA*, and the *trans*-complemented strains SΔ*htrA/HtrA*, SΔ*htrA/HtrA*ΔPDZ, and SΔ*htrA/HtrA*S255A, in a murine model of virulence assessment. **(A)** Survival table of mice infected by sub-cutaneous administration of the various strains. Each experimental group included 4 mice. The resulting Lethal Dose 50% (LD_50_) and the 95% confidence intervals were calculated by global non-linear fit regression using the GraphPad Prism software. The statistical significance of the calculated LD_50_ values was determined by the extra-sum-of-squares F comparison test using the GraphPad Prism software; ^∗^*p* < 0.05. **(B)** Kaplan–Meier survival chart of selected experimental groups, as indicated in the boxed legend. Note that 10^4^ bacteria of the SΔ*htrA* or SΔ*htrA/HtrA*S255A strains were administrated to mice, underlying the virulence attenuation of these strains; only 10^2^ SΔ*htrA/HtrA*ΔPDZ bacteria were administered underlying the high virulence exhibited by the SΔ*htrA* strain upon *trans*-complementation with the ΔPDZ form of HtrA_BA_.

HtrA plays an essential role in *B. anthracis* virulence (Chitlaru et al., 2011b, 2016, 2017), as demonstrated by the observation that Δ*htrA* strains are significantly less virulent than their isogenic parental strains. Disruption of the *htrA* gene results in attenuation of virulence unmatched by any mutation in *B. anthracis*, other than those affecting the bacterial toxins LT and ET (Chitlaru et al., 2016), in all rodent animal models of anthrax. This impact of *htrA* gene disruption on virulence, underlines the importance for anthrax pathogenesis of the ability of the bacteria to cope with the hostile environment, encountered in the infected host (see section “Introduction”). In the current study, by using an SC murine infection assay of *B. anthracis* virulence (Chand et al., 2009), we probed the importance of the protease and PDZ domains in enabling HtrA_BA_ to exert its role in *B. anthracis* virulence. While the model of choice for *B. anthracis* Sterne-strain infection is constituted by respiratory exposure of mice (or guinea pigs) to spores, sub-cutaneous administration of vegetative cells to mice provides a virulence model exhibiting an extended window of infection doses. Consequently, the ICR-outbred murine model of SC exposure was adequate for evaluation of the impact that various genetic manipulations and/or heterologous gene expression (such as *trans*-complemented strains) on the virulence of various strains (see for example Chitlaru et al., 2016, 2017). Thus, *B. anthracis* Sterne Δ*htrA* cells *trans*-complemented with the FL, ΔPDZ or S255A forms of HtrA_BA_ were used side-by-side with the WT Sterne and the Sterne Δ*htrA* strains for determination of their LD_50_ values in mice (Figure 6). All animals exposed to the Sterne strain succumbed 3–6 days post inoculation (LD_50_ = 10 CFU) while significant survival was demonstrated by the animals inoculated with the Sterne Δ*htrA* strain (LD_50_ = 10^3^ CFU), in line with previous experiments in the murine system (Chitlaru et al., 2016). As expected, *trans*-complementation with the FL form of HtrA fully restored the virulence of the bacteria, as attested by an LD_50_ value of 10 CFU, undistinguishable of that exhibited by the WT parental Sterne strain. Interestingly, the same full complementation effect was mediated by expression of the ΔPDZ form of HtrA, but not by expression of the S255A form (Figure 6). Thus, it may be concluded that the proteolytic activity of HtrA is essential for *B. anthracis* virulence while the PDZ is dispensable for its role.

## Discussion

HtrA_BA_ is an important virulence determinant of *B. anthracis* (Chitlaru et al., 2011b). Owing to the very high virulence attenuation associated with its disruption (6 orders of magnitude increase in the LD_50_ value compared to the parental strain in the guinea pig model, Chitlaru et al., 2016), which does not affect the synthesis and secretion of the *B. anthracis* Protective Antigen (PA, necessary for the elicitation of protective immunity), *B. anthracis* Δ*htrA* strains were recently shown to represent efficacious next generation live attenuated anthrax vaccine (Chitlaru et al., 2016, 2017). It is interesting to note that HtrA_BA_ is a unique mono-cistronic gene, unlike the situation in *B. subtilis* which possess 2 highly homologous genes, HtrA (the product of the *ykdA* gene) and HtrB (the product *yvtA* gene) which may mutually compensate when the function of one of them is abrogated (Hyyrylainen et al., 2001; Noone et al., 2001; Darmon et al., 2002). Such a compensatory mechanism is not active in *B. anthracis*, therefore disruption of HtrA_BA_ is associated with a well-defined phenotype. Yet, the *B. anthracis* Δ*htrA* strains are not affected in their *in vitro* growth and appear to manifest their phenotype in conditions which are reminiscent of the *in vivo* infection (Chitlaru et al., 2011b). As many other bacterial HtrA paralogs, HtrA_BA_ is composed of a signal peptide, *trans*-membrane domain, a trypsin-like protease catalytic domain, and a C-terminal PDZ domain. In the study documented in the current report we interrogated the relative impact that the proteolytic activity (mediated by the catalytic trypsin-like protease domain) and the PDZ domain (necessary for the chaperone activity) have in manifestation of the various functions mediated by HtrA_BA_. Based on *trans*-complementation of Δ*htrA* strains with either a wild-type, truncated (ΔPDZ), or non-proteolytic form (S255A) of HtrA_BA_, or by inspection of strains exhibiting modified chromosomal alleles of HtrA_BA_, it is shown that the proteolytic activity is necessary for the N-terminus processing and subsequent secretion of HtrA_BA_, for the role of HtrA_BA_ in resilience of the bacteria to heat, oxidative and salt stress, for the role in the biosynthesis of the S-layer and is essential for manifestation of the role of HtrA_BA_ in virulence, while the PDZ domain is dispensable for all these roles. On the other hand, the proteolytic activity appears to be dispensable for the HtrA-mediated induction of the NprA protease under limited nutrient conditions.

### Self-Processing and Secretion of HtrA_BA_

One of the major observations of this study is that proteolytic active forms of HtrA_BA_ (either the full-length or the ΔPDZ versions) undergo N-terminal proteolytic self-processing which results in the removal of a highly hydrophobic putative *trans*-membrane domain and consequently in the release of HtrA_BA_ as a secreted protein (Figure 2). By LC-MS peptide mapping of the autolytic processed and un-processed forms of HtrA_BA_, we were able to map the putative N-terminal cleavage site to the methionine residue at position 103. Removal of the N-terminal domain of HtrA_BA_ by self-processing in the course of its secretion is suggested also by the recent observation of Pomerantsev et al. (2017) who documented that a shorter than expected form of over-expressed HtrA was released from an engineered *B. anthracis* protease-free strain (exhibiting deletion of 10 various proteases). The boundary of the removed N-terminal segment was mapped at position 76 of the protein, about 23 amino acids up-stream of the cleavage site implied by our data. This inconsistency may stem from the differences in HtrA expression level of the systems used in the two studies or (alternatively but not mutually exclusive) from the fact that different *B. anthracis* strains were used in the two studies; interestingly, one simple interpretation which may reconcile the discrepancy is that an additional proteolytic activity absent from the Ames BH500 non-proteolytic engineered strain (Pomerantsev et al., 2011, 2017) but present in the ΔVollum strain employed in the current study, may be involved in the processing of the N terminus of HtrA, in conjunction with the autolytic activity. In any case, while this issue awaits to be resolved, both studies bring evidence that the removal of a highly hydrophobic N-terminal region, downstream of the canonical export signal sequence, is an essential step for the secretion of HtrA.

The mechanism by which HtrA_BA_ undergoes both removal of the N-terminal canonical export-signal peptide and autolytic processing of the N-terminus represents a matter of further study. Interestingly, the autolytic unprocessed HtrA_Ba_ (which prevails in the case of the proteolytically deficient S255A mutant and consequently fails to be secreted, Figure 2) harbors an intact N-terminus, indicating that its signal peptide was not removed (see LC-MS mapping peptide coverage of HtrA_BA_ in Figure 3A,B). The fact that the S255A mutation prevents not only the N terminus autolytic processing but also the removal of the HtrA_BA_ canonical Sec-pathway signal peptide by a putative peptidase (Auclair et al., 2012; Tsirigotaki et al., 2017) may indicate that the two processes occur in concert or that they are mechanistically connected. Theoretically, the two processes are redundant and independent, and removal of the signal peptide by a putative peptidase may have been sufficient to disrupt the hydrophobic domain and consequently to release the protein from its membrane form. Therefore it is possible that N-terminal self-processing may be important for other aspects of HtrA function, in addition to its secretion.

N-terminal autolytic cleavage of HtrA has been observed in several other serine proteases of the HtrA family. The *E. coli* DegP serine protease undergoes N-terminal autolytic degradation (Skórko-Glonek et al., 1995) provided that its proteolytic catalytic triad is intact. In the case of DegP, the autolytic process appeared to be mainly necessary for the control and modulation of the quaternary/oligomeric structure of the protein, rather than for its secretion: N terminus self-proteolysis of DegP results in the removal of two cysteine residues which are involved in stabilization and multimerization of the molecules via disulfide bridges (Skórko-Glonek et al., 2003). Of note, *B. anthracis* HtrA N-terminus does not contain any cysteine residue and furthermore, it does not appear to be involved in determining the general architecture of the monomeric molecule (see Figure 3C). The *Mycobacterium tuberculosis* HtrA2 protein (Mtb HtrA2), one of the three HtrA-like serine proteases harbored by this bacterium, was also suggested to be released into the medium via an autolytic process, yet in this case, the protein does not exhibit a recognizable secretion peptide sequence (MohamedMohaideen et al., 2008). Furthermore, the autolytic process of Mtb HtrA2 targets additional sites resulting in the generation of short regulatory peptides which are incorporated in the binding cavities of the proteolytic and PDZ domains of the molecule. A similar situation does not seem to occur in HtrA_BA_, based on the observation that no additional proteolytic products of the protein were detected by Western blot analysis. An additional case of amino-terminal self-processing of HtrA was recently reported in *H. pylori* (Albrecht et al., 2018). *H. pylori* HtrA (HbP HtrA) is considered to play an important role in virulence, attributed to its proteolytic activity, specifically targeting host E-cadherin (Hoy et al., 2010). Subsequently, this HtrA-mediated mechanism of virulence was demonstrated in additional Gram-negative gastrointestinal pathogens (Hoy et al., 2012; Abfalter et al., 2016). Autolysis of HbP HtrA appears to have a dual role: on the one hand it is necessary for secretion of the protein. On the other hand, N-terminus autolytic processing affects cleavage of the E-cadherin substrate by modulating the oligomerization state of membrane-bound HbP HtrA which is essential for its proteolytic activity. Recently, the HtrA-like protease from the thermophilic *Brevibacillus* sp. *WF146* strain was shown to be released from the cell surface via autoprocessing of its N-terminal membrane anchor (Zhu et al., 2017), yet interestingly, in this case the autoprocessing required the association of the PDZ domain of the protein with a denatured proteolysis substrate. A similar dependence of the HtrA autolytic process to association of the PDZ domain with substrates was demonstrated in *Magnetospirillum magneticum* HtrA-like protein MamE (Hershey et al., 2016), yet in this case the issue of secretion was not addressed. A similar mechanism linking substrate recognition to auto-proteolysis does not appear to occur in *B. anthracis* in which the N-terminal processing occurs efficiently with truncated ΔPDZ forms of HtrA_BA_ (Figure 2 and see below). Taken together, these studies indicate that N-terminal self-processing is a common characteristic of HtrA serine proteases yet the role of self-processing may differ from case to case. We propose that in the case of HtrA_BA_, the major role of N-terminal autolysis is secretion of the protein. In line with this concept, we have documented in the past that HtrA_BA_ is abundantly secreted *in vivo* (coinciding with expression of the anthrax toxins), can be detected in the circulation of infected animals and consequently can serve as an early biomarker for *B. anthracis* infection (Chitlaru et al., 2006; Sela-Abramovich et al., 2009).

### The Proteolytic Activity of HtrA_BA_ Is Essential While Its PDZ Domain Appears to Be Dispensable for Most of Its Functions

The proteolytic activity of HtrA_BA_, abrogated by the S255A mutation, is demonstrated to be essential not only for autolytic processing, as explained above, but also for the role of HtrA_BA_ in heat, oxidative, and salt resilience (Figure 3 and Supplementary Figure S3), for its role in the biosynthesis of S-layer (Figure 5A and Supplementary Figure S4) and finally for its role in manifestation of *B. anthracis* virulence (Figure 6). These conclusions are based on the failure of the HtrA S255A form to *trans*-complement the phenotype exhibited by the Δ*htrA* strain. Actually, the only iteration in which the role of HtrA was not affected by the S255A mutation (but rather by the PDZ domain deletion) was induction of the NprA extracellular protease (Figure 5B). This former aspect of HtrA function involves the (direct or indirect) role of HtrA as a stress-related regulatory factor (Chitlaru et al., 2011b), which is not related to its proteolytic ability but rather to a more pleiotropic role of HtrA_BA_ in modulating expression of genes involved in stress response. Accordingly an extensive transcriptomic analysis of HtrA_BA_ mutated strains evidenced HtrA-dependent expression-regulation of a large number of genes under various stress regimens (manuscript in preparation).

The essentiality of the proteolytic activity is a hallmark of all serine proteases of the HtrA family (extensively reviewed by Pallen and Wren, 1997; Kim and Kim, 2005; Clausen et al., 2011; Singh et al., 2011; Hansen and Hilgenfeld, 2013; Skórko-Glonek et al., 2013; Chang, 2016; Backert et al., 2018). In this regard, HtrA_BA_ is not an exception. Yet the dependence on HtrA activity on the integrity of the proteolytic domain is not always as strict as evidenced in this study. For example, in *Campylobacter jejuni* (Bæk et al., 2011), HtrA chaperone activity is sufficient for growth under mild stress conditions. Furthermore, proteolytic inactive forms of *E. coli* DegP (exhibiting an S210A mutation, analogous to the S255A of HtrA_BA_ employed in the current study) were efficient in *trans*-complementing the temperature sensitivity of an *htrA* mutant strain (Skórko-Glonek et al., 2007). These studies suggested that sometimes association of HtrA to misfolded proteins without their degradation may be sufficient for maintenance of stress resilience. This appears not to be the case with HtrA_BA_ in which stress sensitivity of the Δ*htrA* strain could not be alleviated by expression of the S255A variant of HtrA_BA_. As mentioned above, the proteolytic-inactive S255A form of HtrA_BA_ fails to be secreted, therefore additional studies will be required to determine whether the failure of this form to *trans*-complement the Δ*htrA* stress-sensitivity phenotype is due to the loss of proteolytic activity *per se* or as a result of its intracellular retention. Yet, it is conceivable that the role of HtrA_BA_ in stress resilience involves catalytic activities performed intracellularly, such as degradation of mal-folded proteins accumulating under stress, therefore it is not likely that this *trans*-complementation failure can be attributed to its intracellular retention. Furthermore, we note that, in preliminary experiments, the virulence attenuation of the Δ*htrA* strain could not be alleviated *in trans* by co-infection with an HtrA overproducing strain (data not shown) suggesting that secretion of HtrA is not essential for manifestation of its role in virulence.

While the importance of the HtrA_BA_ proteolytic domain, was expected considering the fact that the major role of HtrA is related to the quality control of proteins and degradation of mal-folded proteins which may accumulate under stress conditions, our data show, that the PDZ domain of HtrA_BA_ is dispensable for many of its functions (see below) and most importantly for manifestation of the *B. anthracis* virulence. This observation is presumably, the most unexpected out-come of this study and is most-intriguing in light of the extensive studies carried out with similar proteins which demonstrated the pivotal role of the PDZ domain for their function. For example, in contrast to our observations with HtrA_BA_, studies of various mutated variants of *Salmonella enterica* (S. Typhimurium) HtrA established that absence of either one of the 2 PDZ domains present in this protein completely abolished the ability of HtrA to complement the growth defects of an *htrA* mutant (Lewis et al., 2009). A similar situation was reported in *Legionella pneumophila* HtrA, although in this case, *trans*-complementation of the intracellular replication defect of an HtrA mutant, could be partially promoted by a truncated variant containing only the first PDZ domain and completely abolished only by deletion of both PDZ domains (Pedersen et al., 2001). Analogous observations supporting the essentiality of the PDZ domain were documented in *E. coli* DegP in which deletion of its both PDZ domains completely abolished the protease activity of the protein and expression of such a truncated form in an *htrA* mutant could not complement its temperature sensitive phenotype (Spiess et al., 1999). Similarly to *Legionella* HtrA, only the first PDZ domain of DegP emerged as absolutely essential for the activity of HtrA (Iwanczyk et al., 2007).

It is possible that the dispensability of the PDZ domain of HtrA_BA_ reflects an inhibitory role of the PDZ domain on the proteolytic activity, such as suggested for DegS, the *E. coli* stress response protease and an archetype of bacterial serine proteases of the HtrA family. DegS was shown to exist in a proteolytic-inactive form in which the PDZ domain allosterically prevents the activity of the protease domain by restricting the access to the protease active site (Wilken et al., 2004). The PDZ domain was demonstrated to bind peptides derived from mal-folded proteins accumulating during hostile environmental conditions, and consequently to relieve the allosteric inhibition resulting in activation of the proteolytic activity (Walsh et al., 2003; Wilken et al., 2004). If that was the case also in HtrA_BA_, the deletion of PDZ domain would cause perpetuation of the proteolytic active conformation resulting in the ability of the ΔPDZ truncated form to *trans*-complement the Δ*htrA* phenotype, as indeed recorded in our study. Yet, the analogy between DegS and HtrA_BA_ is only limited, since, unlike the situation in *B. anthracis*, a truncated form of DegS lacking the PDZ domain exhibited only marginal levels of proteolytic activity (Walsh et al., 2003) suggesting that the DegS PDZ domain may have additional positive roles on the overall activity of DegS. It is interesting to note that a similar dispensability of the PDZ domain has been reported only for the human proteases HtrA1 and HtrA3, representing eukaryotic paralogs of bacterial quality-control serine proteases of the HtrA family, in which it appears that activity is not affected by deletion of their respective PDZ domains (Truebestein et al., 2011; Glaza et al., 2015).

## Conclusion

Here we presented for the first time a study addressing the importance of the proteolytic and PDZ domains of *B. anthracis* HtrA for its function and most importantly for manifestation of *B. anthracis* virulence. These data strongly suggest that mutational abrogation of the proteolytic activity of HtrA and truncation of its PDZ domain, have different consequences on the activity of the protein. The major conclusions of the study are (i) the proteolytic activity of HtrA_BA_ is essential for its autolysis and secretion, for the role of HtrA in stress resistance and for manifestation of *B. anthracis* virulence, and (ii) the unique PDZ domain is dispensable for all these processes. Accordingly, HtrA_BA_ exhibits significant differences compared to other bacterial serine proteases of the HtrA family. The observations documented in this report are relevant to the design of novel therapeutic strategies targeting *B. anthracis* HtrA.

## Author Contributions

TC conceptualized the study. MI, SR, UE, HC, TC, AB-K, and AT performed experiments. SR performed statistical analysis of data. TC, MI, and OC conceived, coordinated, and supervised the study. TC, AB-K, and OC analyzed and interpreted the data. TC wrote the manuscript. All authors revised and agreed on the manuscript.

## Conflict of Interest Statement

The authors declare that the research was conducted in the absence of any commercial or financial relationships that could be construed as a potential conflict of interest.
