# Supplementary material for: Distinct Contribution of the HtrA Protease and PDZ Domains to Its Function in Stress Resilience and Virulence of Bacillus anthracis
Source: Front Microbiol. 2019 Feb 18;10:255. doi: 10.3389/fmicb.2019.00255 (PMC6387919; doi:10.3389/fmicb.2019.00255)
Supplement: Supplementary file 1 [file Data_Sheet_1.pdf]

Distinct Contribution of the HtrA Protease and PDZ Domains to its Function in Stress Resilience and Virulence of *Bacillus anthracis*

Ma'ayan Israeli, Uri Elia, Shahar Rotem, Hila Cohen, Avital Tidhar, Adi Bercovich-Kinori, Ofer Cohen, Theodor Chitlaru

Supplementary Material

Supplementary Table S1

E. coli bacterial strains, plasmids and oligonucleotide primers used in this study.

Supplementary Figure S1

Expression of the trans-complementation forms of $HtrA_{BA}$ (HtrA FL, HtrAΔPDZ and HtrAS255A) in *E coli*, purification and functionality assays.

Supplementary Figure S2

Schematic representation of the chromosomal $HtrA_{BA}$ locus and of the strains exhibiting modified chromosomal $HtrA_{BA}$ alleles.

Supplementary Figure S3

Growth of the WT parental ΔV, ΔVΔ*htrA*, and the trans-complemented strains ΔVΔ*htrA/HtrA*, ΔVΔ*htrA/HtrA*ΔPDZ and ΔVΔ*htrA/HtrA*S255A at 37°C and 42°C.

Supplementary Figure S4

Expression of S-layer proteins by the *B. anthracis* parental WT ΔV, the ΔVΔ*htrA* and the ΔV strains exhibiting modified *htrA* alleles ΔV$htrA_{\Delta PDZ}$ and ΔV$htrA_{S255A}$, as well as the trans-complemented strains ΔVΔ*htrA/HtrA*, ΔVΔ*htrA/HtrA*ΔPDZ and ΔVΔ*htrA/HtrA*S255A.

Supplementary TABLE S1 | *E. coli* bacterial strains, plasmids and oligonucleotide primers used in this study. *B. anthracis* strains are detailed in Table 1.

E. coli **Strain**	Description	Source
DH5α	*endA1 recA1*	Bioline
BL21 (DE3)	15::Kan (DE3)	Novagen
dam⁻dcm⁻	Dam13::Tn9(Cm^R)dcm-6	NEB
**Plasmids**	Description	Reference
pASC-α	pASCα-derived PA expression vector under *amy* promoter	Chitlaru et al., 2011
pASC-α HtrA	pASC-α htrA expression vector with C' 6*His tag between *SnaB*I and *BamH*I sites	This study
pASC-α N'Htra	pASC-α N'htrA (containing nucleotides 1-900 of the *htrA* gene) expression vector with C' 6*His tag between *SnaB*I and *BamH*I sites	This study
pASC-α $HtrA_{S255A}$	Protease mutant *htrA* expression vector with C' 6*His tag between *SnaB*I and *BamH*I sites	This study
pEGS-cya	Allelic replacement vector for *cya* deletion	Levy et al 2012
pEGS-N'HtrA	Allelic replacement vector for deletion of C'htrA (900-1242bp)	This study
pEGS-$HtrA_{S255A}$	Allelic replacement vector for mutation of HtrA protease active site ($htrA_{S255A}$)	This study
pXX-IsceI	PXX- I sceI endonuclease expression vector, Km^R	Levy et al 2012
pET-21b	Amp^R, *E.coli* expression vector	Novagen
pET-21b-HtrA	pET-21b expression vector with HtrA at *EcoRI* and *XhoI* sites	This study
pET-21b-N'HtrA	pET-21b expression vector with N'HtrA (1-900bp) at *EcoRI* and *XhoI* sites	This study
pET-21b-$HtrA_{S255A}$	pET-21b expression vector with $HtrA_{S255A}$ at *EcoRI* and *XhoI* sites	This study
**Primer***	**sequence**	**Use**
BA531	agacct**agatct**tatacaaaaaggagtacgtatATGGGATATTACGACGGACCA	Cloning of htrA-6XHis
BA819C	agatagacct**ggattc**ttaatggtgatggtgatggtgTTGATTCTTTGTAGCTGAGTTATCT	Cloning of htrA-6XHis

BA818	TGAGAAGAAAAAAGTAGGCGAGAAAG	Sequencing of EC1489
BA820C	**ggattc**ttaatggtgatggtgatggtg	PCR detection of transformants
BA821C	agatagacct**ggattc**ttaatggtgatggtgatggtgTTTTTCAAGTGATTCAATAACTGGT	Cloning of N'htrA-6XHis
BA822	ATAAGCGTGCAGATTGGAATGCTCAA	PCR detection of transformants
BA825C	CCCGATTTTCTCACTTCTCTCG	Sequencing of EC1491
BA826	GGTGCTAATGTTAATAAAGTTGCC	Sequencing of EC1491
BA827	agatacct**GCGGCCGC**ATGGGATATTACGACGGACCAA	Cloning of EC1495 and EC1496
BA828C	gtcctc**ACTAGT**TTATTTTTCAAGTGATTCAATAACTGG	Cloning of EC1495
BA829	gtcctc**ACTAGT**TCGGAATAAAAGAAAGTCTCT	Cloning of EC1495
BA830C	agacct**GGCGCGCC**TCTTCATTATATTGGAACTGAT	Cloning of EC1495
BA831C	agacct**GGCGCGCC**TTATTGATTCTTTGTAGCTGAGT	Cloning of EC1496
htrA-pET-for-EcoRI	gcaaatgggtcgggatcc**gaattc**ATGGGATATTACGACGGACCA	Cloning of HtrA to pET-21b
htrA-pET-rev-Xho-S	cctttcgggctttgttagcagccggatc**ctcgag**TTAATGGTGATGGTGATGGTG	Cloning of HtrA to pET-21b

* The homology region to the coding sequence is marked in capital letters while homology to vector-derived sequences are in lowercase letters. The restriction sites used for the cloning of the corresponding PCR fragment are in bold.

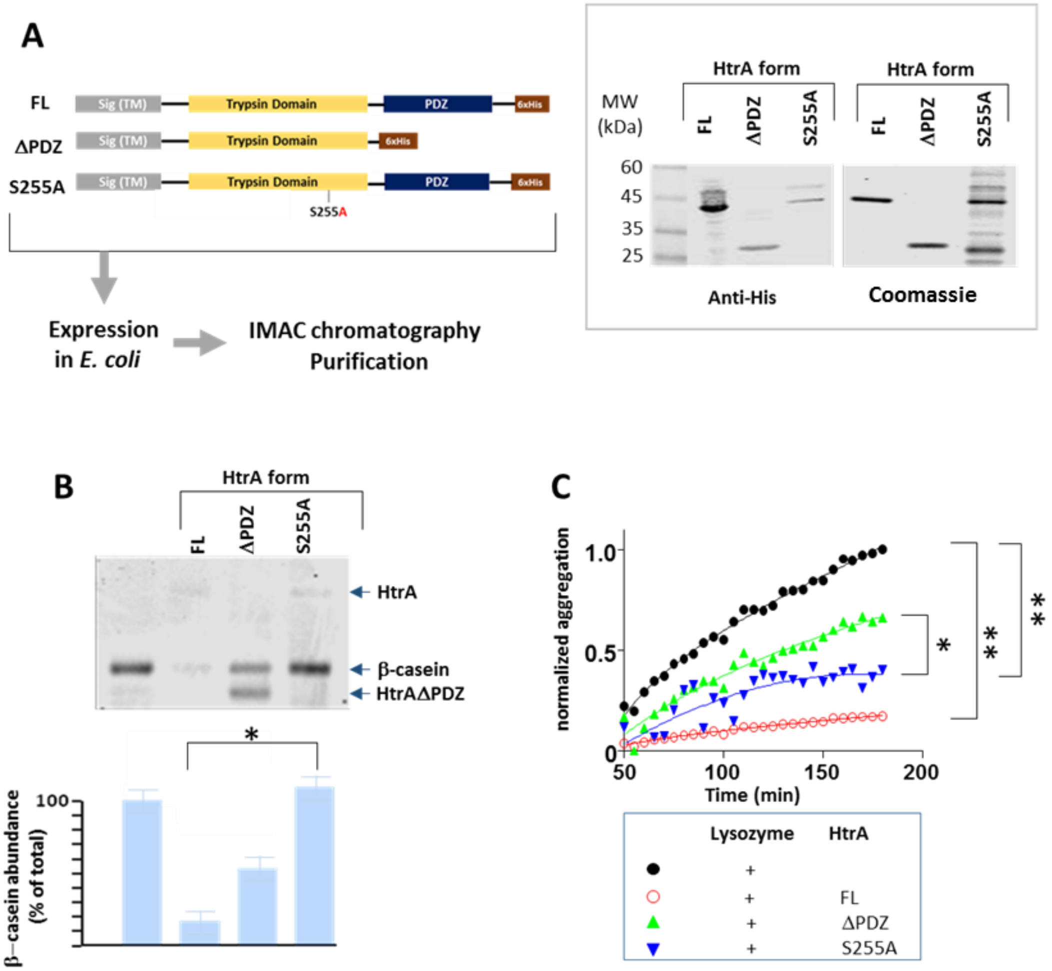

Supplementary Figure S1 | Expression of the trans-complementation forms of HtrA$_{BA}$ (HtrA FL, HtrAΔPDZ and HtrAS255A) in *E coli*, purification and functionality assays. **(A)** Schematic representation of the HtrA forms expressed in E. coli. Right boxed panel: Western blot analysis of purified forms of HtrABA following IMAC (immobilized metal affinity chromatography). While the full-length and the ΔPDZ forms of HtrA were obtained in highly enriched fractions by IMAC, the HtrAS255A form could not be purified to homogeneity, as attested by the commassie-blue stained gel. Note that the additional bands do not react with the anti-his tag or anti-HtrA antibodies, establishing that they do not represent degraded forms of HtrA. The identity of these proteins and the reason for their co-elution only with the His-tagged HtrAS255A form but not with the FL or ΔPDZ forms, was not further studied. **(B)** β-Casein proteolysis assay using the different forms of HtrA$_{BA}$. Upper panel depicts the SDS-PAGE of the reactions, lower panel represents scan quantification of the β-casein bands from the gel, using an Odyssey CLx scanner (Li-Cor Biosciences,Lincoln, NE, USA) and analyzed by the Li-Cor Image Studio (ver. 5.2). Note the complete loss of proteolytic activity by the S255A form of HtrA$_{BA}$;*$p<0.05$, by student t-test. **(C)** Lysozyme aggregation assay in the presence/absence of the various HtrA$_{BA}$ forms. Note that the ΔPDZ form is significantly more affected in its ability to prevent aggregation of the substrate, in line with the notion that the PDZ domain is required for the optimal chaperone activity of HtrA. Statistical significance was determined by a global non-linear regression analysis using the GraphPad Prism 5.0 software (GraphPad Software, San-Diego, CA, USA); *$p<0.05$, **$p<0.01$.

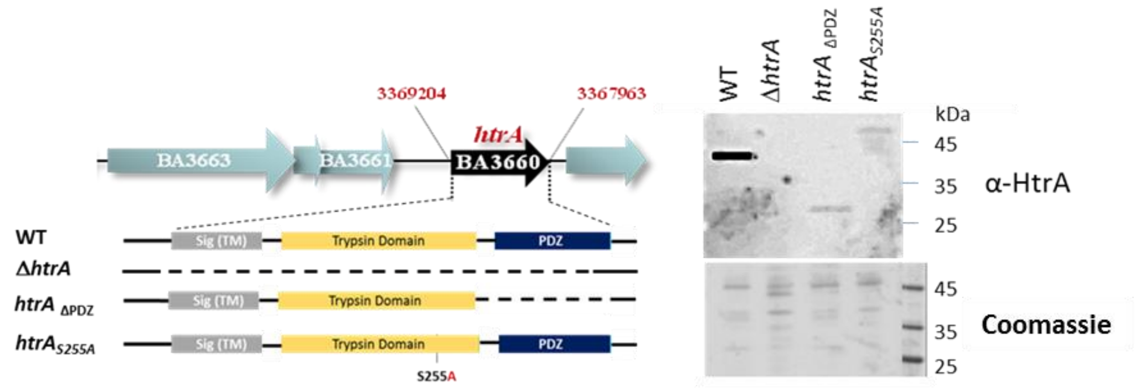

Supplementary Figure S2 | Schematic representation of the chromosomal HtrA$_{BA}$ locus and of the strains exhibiting modified chromosomal HtrA$_{BA}$ alleles. Modification of the *B. anthracis* chromosomal *htrA* gene enables comparison between *B. anthracis* wild-type, *B. anthracis* Δ*htrA* and novel strains containing an *htrA* truncated allele (*htrA*$_{\Delta PDZ}$) or a mutated allele (*htrA*$_{S255A}$). HtrA$_{BA}$ is encoded by the indicated mono-cistronic gene (NCBI locus BA3660). The position of the locus in the *B. anthracis* Ames ancestor genomic sequence is indicated. Right panel: Western-blot analysis using anti-HtrA antibodies of cellular extracts of the 4 strains described in the left scheme. Note that the HtrA form expressed in the *htrA*$_{S255A}$ strain, exhibits a slower electrophoretic migration due to its failure to be auto-lytically processed (see Section 3.2).

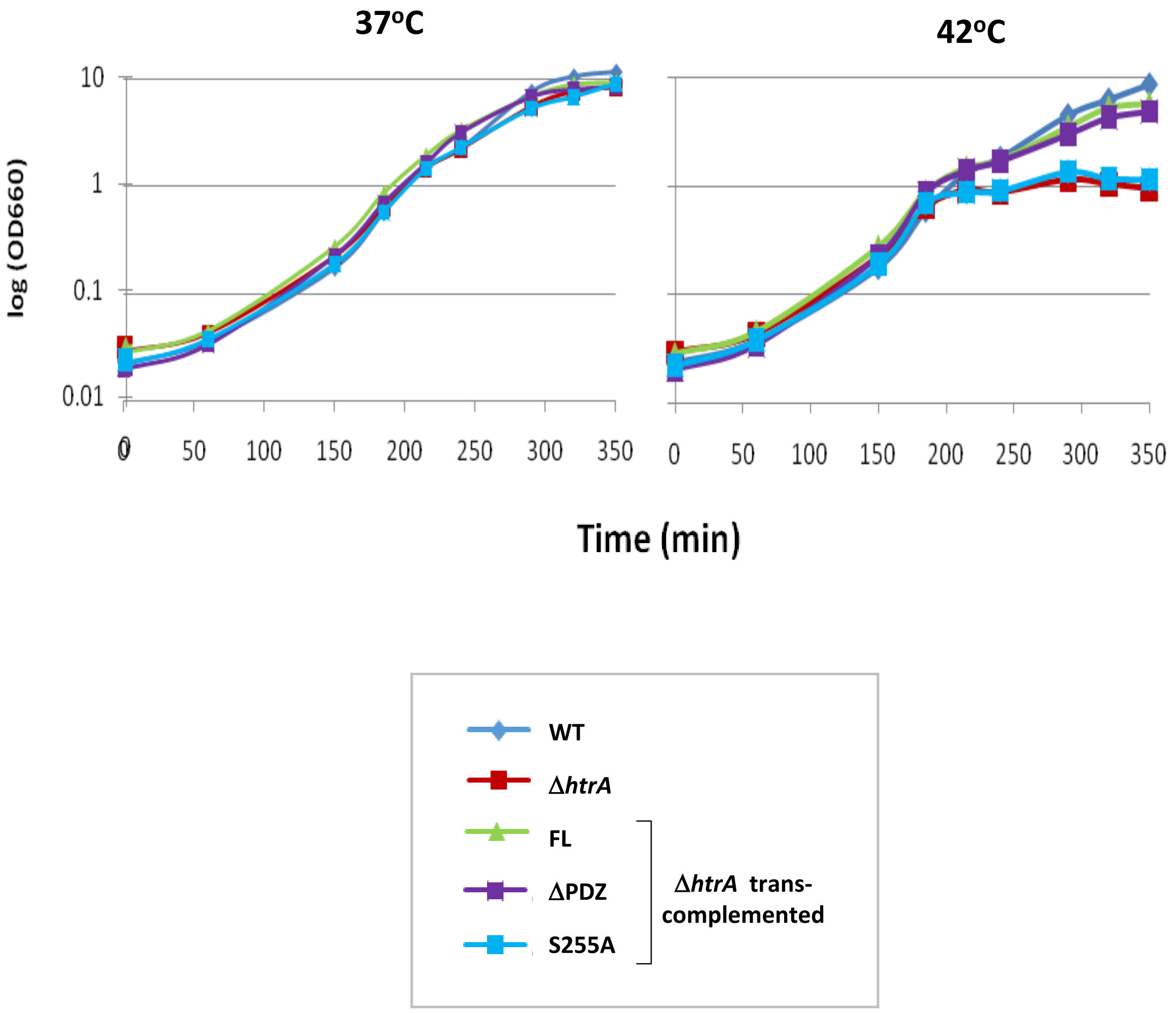

Supplementary Figure S3 │Growth of the WT parental ΔV strain, ΔVΔ*htrA*, and the trans-complemented strains ΔVΔ*htrA/HtrA*, ΔVΔ*htrA/HtrA*ΔPDZ and ΔVΔ*htrA/HtrA*S255A at 37°C and 42°C. The various strains were grown in BHI medium. Over-night cultures of the 5 strains (OD= 10-13) were used to inoculate fresh BHI at a starting OD of 0.05. At the indicated time, when the cultures were in the mid-log phase (OD=1), the cultures were split into two twin flasks which were incubated at 37°C or 42°C.

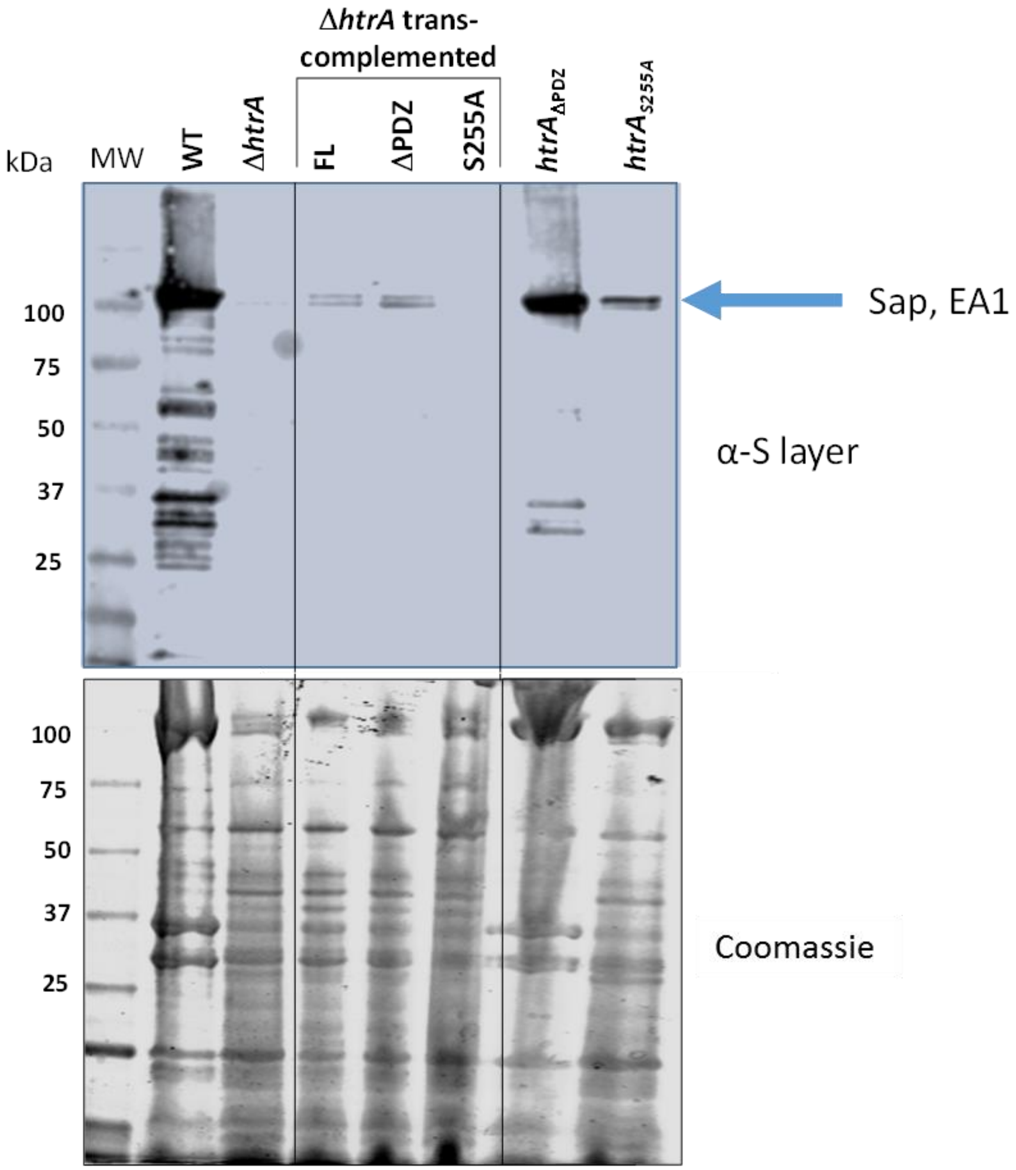

Supplementary Figure S4 │Expression of S-layer proteins by the *B. anthracis* parental WT ΔV, the ΔVΔ*htrA* and the ΔV strains exhibiting modified *htrA* alleles ΔV*htrA*$_{\Delta PDZ}$ and ΔV*htrA*$_{S255A}$, as well as the trans-complemented strains ΔVΔ*htrA/HtrA*, ΔVΔ*htrA/HtrA*ΔPDZ and ΔVΔ*htrA/HtrA*S255A. The various strains were grown in BHI media for 18 hours. Bacterial cells were washed in PBS and boiled in SDS-PAGE buffer for 15 minutes before analysis. Equal cell equivalent volumes (approx.. 10 OD) of the 5 cultures were loaded for Western blot analysis using antibodies specific for both the Sap and EA1 S-layer proteins. See and **Table 1, Figure 1** and **Supplementary Figure S2** for details regarding the various strains exhibiting modified HtrA$_{BA}$ allels and Δ*htrA* strains trans-complemented with different forms of HtrA$_{BA}$.
